# Online mindfulness-based cognitive therapy as an adjuvant-treatment for Chinese patients with psoriasis: A randomized controlled trial

**DOI:** 10.1016/j.jdin.2026.02.003

**Published:** 2026-02-20

**Authors:** Yan Zhao, Yamei Li, Yunbo Zhang, Mingxin Yan, Qingxuan Du, Jinjin Huang, Fei Li, Dan Han, Yan Zhou

**Affiliations:** a Department of Dermatology, The First Affiliated Hospital of Xi’an Jiaotong University, Xi’an, Shaanxi, China; b Department of Psychiatry, The First Affiliated Hospital of Xi'an Jiaotong University, Xi’an, Shaanxi, China; c Department of Epidemiology and Biostatistics, School of Public Health, Xi'an Jiaotong University, Xi'an, Shaanxi, China; d Center for Occupational Safety and Health, Institute for Hygiene of Ordance Industry, Xi’an, Shaanxi, China; e Department of Cardiology, The Second Affiliated Hospital of Xi'an Jiaotong University, Xi'an, Shaanxi, China

**Keywords:** Anxiety, Depression, Online Mindfulness-Based Cognitive Therapy, Psoriasis Area and Severity Index, Psoriasis

## Abstract

**Background:**

Patients with psoriasis often experience emotional distress, which may be alleviated through psychological interventions.

**Objective:**

To evaluate online Mindfulness-Based Cognitive Therapy (MBCT) as an adjuvant treatment for psoriasis, focusing on severity of lesion, anxiety, depression, quality of life, and itching.

**Methods:**

This randomized trial enrolled 109 patients with psoriasis assigned to either treatment as usual (TAU) or TAU plus MBCT, which included 8 weekly online sessions. Primary outcomes included Psoriasis Area and Severity Index (PASI), Self-Rating Anxiety/Depression Scale (SAS/SDS), and Dermatology Life Quality Index (DLQI). Itching was a secondary outcome measured by Visual Analogue Scale (VAS). Assessments were conducted at baseline, 4, 8, and 12 weeks. Treatment effects were analyzed with mixed linear models.

**Results:**

A total of 109 patients were randomized 1:1 to MBCT + TAU (*n* = 53) or TAU (*n* = 56). Significant group × time interactions favoring MBCT + TAU were observed for PASI (F = 3.746, *P* = .013, d = 0.331), SDS (F = 3.205, *P* = .025, d = 0.269), DLQI (F = 3.130, *P* = .028, d = 0.309), and VAS (F = 3.126, *P* = .028, d = 0.295). No significant between-group difference was found for anxiety.

**Limitations:**

Single-center.

**Conclusions:**

Adjunctive online MBCT led to improvements in psoriasis severity, depression, DLQI and itching, suggesting its potential to enhance comprehensive psoriasis management.

**Capsule Summary**

• This study adds to growing evidence that psychological interventions like MBCT can benefit patients with psoriasis by improving mental health and symptom burden.
• Online MBCT can be integrated into routine care to support patients’ emotional well-being and reduce disease severity, especially where access to in-person therapy is limited.

## Introduction

Psoriasis is a chronic, recurrent, systemic, and inflammatory disease, which is driven by immune-mediated factors and influenced by both genetic and environmental components.^1^^,^^2^ Epidemiological surveys have found the crude prevalence of psoriasis to be 0.59% in China.^3^ Evidence from previous studies indicates that patients with psoriasis are prone to psychiatric comorbidities, exhibiting a higher prevalence of depression than the general population.^4^ Romiti et al reported that 53% of 1125 patients with psoriasis had psychological comorbidities, with anxiety disorders and depression affecting 39.7% and 27.1% of patients, respectively.^5^ Notably, depression in individuals with psoriasis may increase the risk of additional comorbidities. A cohort study found that psoriasis patients with major depressive disorder (MDD) had an increased risk of developing psoriatic arthritis.^6^ Similarly, in individuals with psoriasis, depression was associated with an increased risk of myocardial infarction, stroke, and cardiovascular death, especially among acute depression episodes.^7^ They were more prone to anxiety disorders.^8^ Significant psychiatric issues in psoriatic patients encompass depression, anxiety disorders, suicidal behavior, and suicidal thoughts.^5^ Identifying and addressing comorbid mental health conditions in individuals with psoriasis is crucial for comprehensive disease management, as psychiatric disorders can negatively impact clinical outcomes and overall prognosis.

The 2023 Chinese guidelines recommend managing psoriasis with both physical treatment and psychological or psychiatric support.^9^ Given the limited efficacy of oral antidepressant medications, psycho-behavioral interventions may offer a safer and more effective alternative. Previous research has demonstrated the effectiveness of Mindfulness-Based Cognitive Therapy (MBCT) as a psychological intervention for improving mental health outcomes in patients with various chronic conditions. Originally developed to prevent depression recurrence^10^^,^^11^ MBCT has been increasingly applied to chronic illnesses such as chronic pain^12^ and tinnitus^13^ showing favorable outcomes. Studies show it effectively reduces depression and anxiety in conditions like heart disease^14^ diabetes^15^ and cancer, with improved satisfaction in breast cancer.^16^ MBCT integrates positive stress reduction with principles from cognitive science.^17^ Maddock et al found that MBCT improved mood and well-being by increasing self-compassion, improving attention, and reducing rumination and worry.^18^ Research on MBCT for psoriasis is limited and exploratory; a small pilot study underscores the need for a full randomized trial to assess its effectiveness.^19^

During the COVID-19 pandemic, delays and disruptions in treatment exacerbated anxiety and depression among patients. In this context, there is a critical need for effective and safe adjunctive therapies for individuals with psoriasis. Online MBCT offers a convenient and accessible approach for patients to learn and practice. Therefore, we hypothesized that online MBCT could alleviate anxiety and depression, improve clinical outcomes, and enhance quality of life in patients with psoriasis.

## Methods

### Study design

The study was an open-label randomized controlled trial. The study compared online MBCT plus treatment as usual (TAU) with TAU alone. Ethical approval was granted from the Ethics Committee of the First Affiliated Hospital of Xi'an Jiaotong University (number XJTU1AF2019LSL-027). The study was pre-registered at ClinicalTrials.gov under the registration number NCT05010044.

### Participants

Patients with psoriasis seeking treatment at the First Affiliated Hospital of Xi'an Jiaotong University were invited to participate in the trial, which was conducted from August 2021 to December 2022. Inclusion criteria were: age 18 to 70 years, diagnosis consistent with the Classification Criteria for Psoriasis, normal or corrected-to-normal vision and hearing, and the ability and willingness to provide informed consent. Exclusion criteria included impaired hand dexterity preventing smartphone use, severe mental illness or current use of antipsychotic medication, and pregnancy. Baseline characteristics are detailed in Table I. The sample size was estimated based on the expected 25% difference in PASI 50 response rates between the TAU group (25%) and MBCT + TAU group (50%). Accounting for a dropout rate of approximately 20%, a sample size of at least 53 participants per group provided 80% power to detect this difference at a one-sided type I error rate of 5%.

**Table I:** Sociodemographic, clinical, and psychological characteristics of study sample at baseline

Patient demographics and characteristics	Control group (*n* = 56)	Intervention group (*n* = 53)	*Z/t/χ*^*2*^	*P*
Age years, ($\bar{X}$ ±S)	35.89 ± 11.49	33.79 ± 11.11	0.970	.334
Gender, *n* (%)			2.032	.154
Men	33 (58.93)	24 (45.28)		
Women	23 (41.07)	29 (54.72)		
BMI, ($\bar{X}$ ±S)	23.68 ± 3.48	22.80 ± 4.05	1.221	.225
Course of disease, years, ($\bar{X}$ ±S)	11.61 ± 8.59	10.40 ± 9.09	0.715	.476
Smoking, *n* (%)			0.371	.542
No	39 (69.64)	34 (64.15)		
Yes	17 (30.36)	19 (35.85)		
Drink, *n* (%)			0.031	.859
No	41 (73.21)	38 (71.70)		
Yes	15 (26.79)	15 (28.30)		
Healthy diet, *n* (%)			0.006	.938
No	30 (53.57)	28 (52.83)		
Yes	26 (46.43)	25 (47.17)		
Mental factors, *n* (%)			2.223	.136
No	28 (50.00)	19 (35.85)		
Yes	28 (50.00)	34 (64.15)		
Combined metabolic diseases, *n* (%)			0.001	.985
No	39 (69.64)	37 (69.81)		
Yes	17 (30.36)	16 (30.19)		
Family history, *n* (%)			0.167	.683
No	38 (67.86)	34 (64.15)		
Yes	18 (32.14)	19 (35.85)		
Therapeutic method, *n* (%)			2.090	.148
Traditional treatment	25 (44.64)	31 (58.49)		
Biological preparation	31 (55.36)	22 (41.51)		
Marital status, *n* (%)			0.148	.700
Unmarried	21 (37.50)	18 (33.96)		
Married	35 (62.50)	35 (66.04)		
By age of onset†, *n* (%)			2.215	.137
Early-onset psoriasis	50 (89.29)	52 (98.11)		
Late-onset psoriasis	6 (10.71)	1 (1.89)		
Knowing how to care for skin lesions, *n* (%)			−0.972	.331∗
Not at all	17 (30.36)	21 (39.62)		
Basic	31 (55.36)	26 (49.06)		
Mostly	8 (14.28)	5 (9.43)		
Completely	0 (00.00)	1 (1.89)		
SAS, ($\bar{X}$ ±S)	44.80 ± 8.50	45.80 ± 10.15	−0.248	.805
SDS, ($\bar{X}$ ±S)	48.83 ± 9.32	51.10 ± 10.33	−1.187	.238
DLQI, ($\bar{X}$ ±S)	8.46 ± 5.06	10.15 ± 6.24	−1.287	.201
VAS, ($\bar{X}$ ±S)	1.98 ± 2.36	2.58 ± 2.08	−0.838	.404
PASI, ($\bar{X}$ ±S)	4.74 ± 4.62	6.89 ± 5.00	−1.480	.142

*BMI*, Body mass index; *DLQI*, Dermatology Life Quality Index; *PASI*, Psoriasis Area and Severity Index; *SAS*, Self-Rating Anxiety Scale; *SDS*, Self-Rating Depression Scale; *VAS*, Visual Analogue Score.

∗ Wilcoxon rank test.

† Early-onset psoriasis: beginning age <40 years; late onset psoriasis: beginning age ≥ 40 years.^20^

### Randomization and blinding

Eligible and interested participants were screened for possible additional barriers to participation. After completing consent forms and baseline questionnaires, participants were randomly assigned to either the MBCT + TAU or the TAU group. Participants were randomized with an allocation ratio of 1:1 by an independent researcher using the Research Electronic Data Capture randomization module, which utilized a file created by a random number generator in SAS, version 9.4 (SAS Institute Inc). Due to the nature of psychosocial interventions, blinding of patients and clinicians could not be maintained throughout the study. The researchers responsible for data analysis were blinded to participants’ group assignments. Randomization and intervention delivery were conducted by independent personnel who did not participate in data analysis. All exported datasets were anonymized prior to statistical processing.

### Treatment arms

#### MBCT + TAU

The intervention was based on the standardized 8-week MBCT manual developed by Segal et al.^21^ It was delivered in an online, audio-based format, allowing participants to complete the program at home without geographic constraints. All guided audio materials were prerecorded specifically for this trial by an experienced psychiatrist to ensure uniformity of delivery. The content included core MBCT practices such as body scan meditation, breath awareness, and exercises for observing thoughts as transient mental events. Consistent with prior adaptations of MBCT for chronic physical conditions, minimal psoriasis-specific refinements were incorporated: participants were gently encouraged, when comfortable, to bring mindful awareness to experiences of itch and skin lesions, observing bodily sensations, emotional responses, and urges (eg, the urge to scratch) with openness and non-judgment, without altering the core structure of MBCT. Detailed weekly content and thematic progression are provided in Supplementary Table I, available via Mendeley at https://data.mendeley.com/datasets/jrkd8xjk54/1.

To enhance engagement and minimize attrition, both groups received identical, minimal non-therapeutic weekly telephone contacts. These calls were standardized in frequency, duration, and script and were limited to logistical and motivational support. No therapeutic guidance or mindfulness instruction was provided in either group.

Intervention adherence was predefined based on engagement with the audio-guided MBCT practices; participants practicing fewer than 3 times per week were considered non-adherent. Throughout the study period, TAU for psoriasis was continued in both groups.

#### TAU

The control condition, referred to as TAU, involved standard care, encompassing traditional drug therapy and biological therapy.

### Measures

Primary and secondary outcomes were assessed at 4 time points: baseline, and weeks 4, 8 and 12.

#### Primary outcomes measures

##### Self-Rating Anxiety Scale (SAS) and Self-Rating Depression Scale (SDS)

The SAS and SDS are 20-item self-assessment tools using a 4-point scale, with some items reverse-scored; higher scores indicate greater levels of anxiety or depression.^22^^,^^23^

##### Psoriasis Area and Severity Index (PASI)

The PASI was used as the primary measure of psoriasis severity. It assesses erythema, thickness, and scaling of plaques, together with the extent of body surface involvement across 4 anatomical regions, yielding a total score ranging from 0 to 72, with higher scores indicating greater disease severity.^24^ All PASI assessments were conducted in person during routine outpatient visits and were performed by qualified dermatologists.

##### Dermatology Life Quality Index (DLQI)

The DLQI is a subjective measure of the psychological burden. The DLQI is determined by a 10-item questionnaire. The index ranges from 0 to 30.^25^

#### Secondary outcome measures

The Visual Analogue Scale (VAS) for pruritus ranges from 0 to 10, with higher scores reflecting greater pruritus severity.

### Statistical analysis

Categorical variables were described using frequencies (*n*) and percentages (%), with group comparisons conducted via the χ^2^ test or Wilcoxon rank test, as appropriate. Continuous variables were summarized as means and standard deviations ($\bar{X}$ ±S), with between-group comparisons performed using the independent samples t-test. Linear mixed models were employed to evaluate the differences in changes of SDS, SAS, DLQI, PASI, and VAS scores between the intervention and control groups over time. An intention-to-treat (ITT) analysis was performed as the primary analytic approach to preserve the benefits of randomization and minimize potential bias due to attrition. Missing data in the ITT analysis were handled using linear mixed-effects models under the missing at random assumption. A per-protocol (PP) analysis was additionally conducted as a sensitivity analysis to assess the robustness of the findings among participants with adequate adherence.

Statistical analyses were conducted using International Business Machines (IBM) Statistical Package for the Social Sciences (SPSS) Statistics version 24 and Statistical Analysis System (SAS) version 9.4, with a threshold for statistical significance set at *P* < .05. Effect sizes were calculated as Cohen’s d, with thresholds of 0.2, 0.5, and 0.8 denoting small, medium, and large effect sizes, respectively. Cohen’s d was derived from the F test and computed using the formula d = 2/3 $\sqrt{\left(F/\text{df}\;\right)}$.

## Results

### Participant characteristics

**Fig 1:**
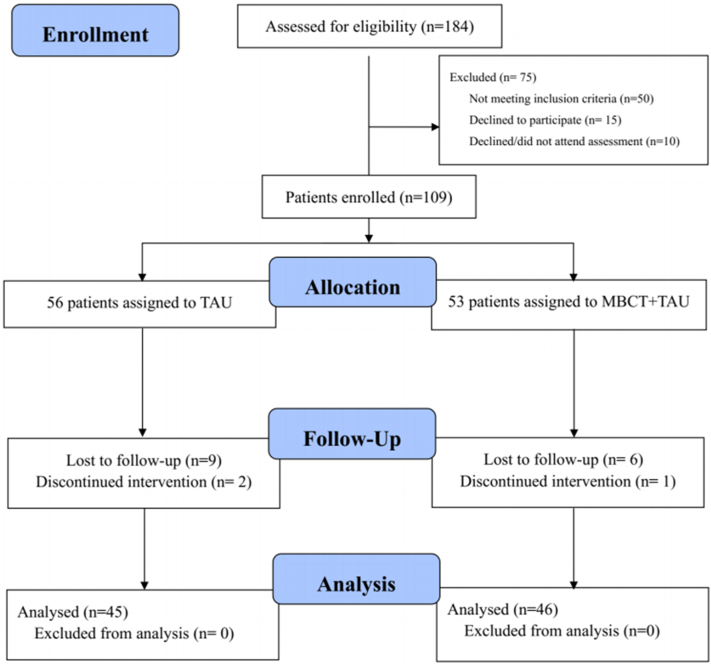
Randomization and follow-up of the patients. *MBCT*, Mindfulness-based cognitive therapy; *TAU*, treatment as usual.

Between August 2021 and December 2022, a total of 184 participants were screened for eligibility. Of these, 75 individuals were excluded: 50 were deemed ineligible, 15 declined to participate, and 10 did not return questionnaires. Consequently, 109 participants were randomly allocated to treatment, with 53 assigned to the MBCT + TAU and 56 to the TAU. Therapy attrition was minimal and not significantly different between groups (odds ratio 1.61, 95% CI 0.57-4.51, *P* = .368); specifically, 11 participants withdrew from the control group, and 7 from the intervention group. The trial profile is illustrated in Fig 1.

Sociodemographic and clinical characteristics of participants are detailed in Table I. There were no significant differences in these characteristics between the groups at baseline.

### Outcomes

#### SDS and SAS

**Fig 2:**
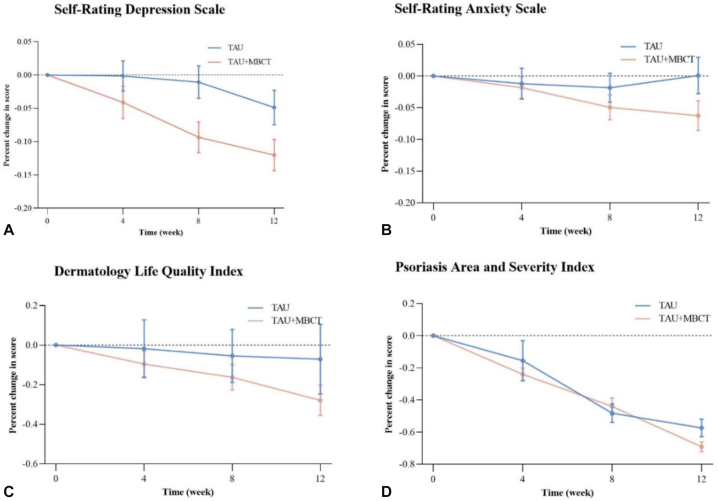
Percent change from baseline on the primary measures between the TAU and MBCT + TAU. Percent change was calculated as (follow-up score − baseline score)/baseline score × 100%. **A,** Self-Rating Depression Scale; **B,** Self-Rating Anxiety/Depression Scale; **C,** Dermatology Life Quality Index; and **D,** Psoriasis Area and Severity Index. I bars indicate the standard error. *MBCT*, Mindfulness-based cognitive therapy; *TAU*, treatment as usual.

Significant time × group effects were observed for SDS (F = 3.205, *P* = .025, Cohen’s d = 0.269, Table II). Compared to the TAU group, the SDS scores in the MBCT + TAU group demonstrated a more pronounced decrease (Fig 2, *A*). However, the results were statistically nonsignificant for time × group effects on SAS (F = 1.136, *P* = .272, Cohen’s d = 0.196, Table II). Notably, the TAU group exhibited a significant increase in SAS scores during the final 8 to 12 weeks of the study, whereas the MBCT + TAU group demonstrated the most substantial decrease in SAS scores during the 4- to 8-week period (Fig 2, *B*).

**Table II:** Mean group differences and effect sizes for primary and secondary outcomes

Measure	Week 0	Week 4	Week 8	Week 12	Effect size (95% *CI*)	Time × Group interaction		
						*F*	*P*	Cohen’s d
SDS						3.205	.025	0.269
Control (*n* = 56)	48.83 ± 9.32	48.17 ± 9.84	47.72 ± 10.64	46.14 ± 11.87	−2.69 (−5.35 to −0.03)	-	-	-
Intervention (*n* = 53)	51.10 ± 10.33	48.35 ± 9.95	45.89 ± 10.84	44.33 ± 10.08	−6.77 (−9.18 to −4.37)	-	-	-
SAS						1.136	.272	0.196
Control (*n* = 56)	44.80 ± 8.50	43.44 ± 7.51	43.26 ± 7.99	43.90 ± 8.94	−0.90 (−3.48 to 1.68)	-	-	-
Intervention (*n* = 53)	45.80 ± 10.15	44.35 ± 8.69	42.84 ± 8.67	42.30 ± 10.31	−3.50 (−5.55 to −1.46)	-	-	-
DLQI						3.130	.028	0.309
Control (*n* = 56)	8.46 ± 5.06	7.38 ± 4.90	7.21 ± 4.67	7.05 ± 4.66	−1.41 (−2.46 to −0.36)	-	-	-
Intervention (*n* = 53)	10.15 ± 6.24	8.26 ± 5.35	6.98 ± 4.69	6.15 ± 5.43	−4.00 (−5.40 to −2.60)	-	-	-
PASI						3.746	.013	0.331
Control (*n* = 56)	4.74 ± 4.62	3.16 ± 3.29	2.03 ± 2.43	1.44 ± 1.94	−3.30 (−4.41 to −2.19)	-	-	-
Intervention (*n* = 53)	6.89 ± 5.00	5.24 ± 3.96	3.69 ± 3.10	2.25 ± 2.37	−4.65 (−5.72 to −3.57)	-	-	-
VAS						3.126	.028	0.295
Control (*n* = 56)	1.98 ± 2.36	1.11 ± 1.28	0.77 ± 1.19	0.89 ± 1.80	−1.09 (−1.70 to −0.48)	-	-	-
Intervention (*n* = 53)	2.58 ± 2.08	1.96 ± 1.52	1.49 ± 1.27	1.09 ± 1.18	−1.49 (−2.02 to −0.96)	-	-	-

*DLQI*, Dermatology Life Quality Index; *PASI*, Psoriasis Area and Severity Index; *SAS*, Self-Rating Anxiety Scale; *SDS*, Self-Rating Depression Scale; *VAS*, Visual Analogue Score.

#### DLQI

Statistically significant time × group effects were found for DLQI (F = 3.130, *P* = .028, Cohen’s d = 0.309, Table II). The MBCT + TAU group exhibited a greater percentage reduction in DLQI scores compared to the TAU group throughout the follow-up period. (Fig 2, *C*).

#### PASI

Significant time × group effects were identified for PASI (F = 3.746, *P* = .013, Cohen’s d = 0.331, Table II). Overall, the reduction in the percentage change in PASI scores was more pronounced in the MBCT + TAU group compared to the TAU group. The TAU + MBCT group showed a greater reduction at week 4 and a more pronounced reduction than the TAU group by week 12 (Fig 2, *D*).

#### VAS

Statistically significant time × group effects were observed for VAS (F = 3.126, *P* = .028, Cohen’s d = 0.295, Table II). Between weeks 0 and 8, the TAU group showed a slightly greater decline in VAS scores; however, from weeks 8 to 12, the MBCT + TAU group demonstrated a more rapid and pronounced reduction (Supplementary Fig 1, available via Mendeley at https://data.mendeley.com/datasets/jrkd8xjk54/1).

#### Sensitivity analysis

The PP analysis included participants who completed the study and yielded results consistent with those of the ITT analysis. Detailed results are provided in Supplementary Tables II and III, available via Mendeley at https://data.mendeley.com/datasets/jrkd8xjk54/1 and Supplementary Figs 2 and 3, available via Mendeley at https://data.mendeley.com/datasets/jrkd8xjk54/1.

### Adverse events

No adverse events were reported in either group.

## Discussion

The findings suggest that online MBCT as an adjuvant treatment for psoriasis may contribute to improvements in treatment outcomes, depression scores, and quality of life compared to medication alone. These potential benefits were observed throughout the intervention period and persisted 1 month later. Notably, no significant difference in anxiety scores was observed between the groups. Similar results were reported in Chronic Obstructive Pulmonary Disease (COPD), which indicate that MBCT primarily alleviates stress by reducing depression rather than anxiety, which aligns with our findings.^26^, ^27^, ^28^

MBCT has gained increasing recognition across a range of fields, including the management of treatment-resistant depression, breast cancer, chronic pain, and functional dyspepsia.^16^^,^^29^, ^30^, ^31^ To date, only a handful of studies have discussed the use of MBCT in psoriasis. A pilot study of 18 psoriasis patients found quality of life improved with MBCT, but anxiety and depression scores showed no significant change, likely due to small sample size.^19^ Similarly, a UK study found that CBT improved quality of life in individuals with psoriasis.^32^

Psoriasis patients receiving online MBCT may experience a greater reduction in SDS scores compared to controls, indicating a potential antidepressant effect. The impact of depression relief on the treatment of psoriasis should not be underestimated. First, it plays a role in reducing the recurrence of psoriasis.^33^ Second, it can lower the body's stress response and decrease the release of inflammatory factors, contributing to symptom relief.^34^ Third, it may alleviate the disease’s impact on patients’ daily life and work, resulting in broader benefits for their families and society.

Our study also revealed the impact of online MBCT on PASI and DLQI scores, suggesting that online MBCT improves patient outcomes and treatment satisfaction, builds a positive doctor-patient relationship, and thus enhances patient compliance. In this context, the positive effects of online MBCT observed in our study may provide a practical approach for implementing the psychological interventions, recommended by the Chinese psoriasis guideline. Our findings can provide a reference for other countries to draw on the Chinese psoriasis guidelines, thereby optimizing patient-centered outcomes.

Beyond statistical significance, the clinical relevance of our findings is supported by effect sizes and established clinical thresholds. The between-group differences showed moderate effect magnitudes, consistent with adjunctive psychological interventions, indicating meaningful incremental benefits rather than a stand-alone therapeutic effect. The MBCT + TAU group achieved a 4.00-point reduction in DLQI scores, meeting the established Minimal Clinically Important Difference (MCID) of 4 points,^35^ whereas the TAU group improved by only 1.41 points. Furthermore, the MBCT + TAU group demonstrated a 6.77-point reduction on the SDS, surpassing the 6.47-point threshold previously identified as clinically meaningful.^36^ Together, these data underscore the tangible clinical impact of the intervention.

Additional finding showed itching reduced more effectively in the intervention group. Basic research suggests that the mechanisms underlying itch and chronic pain exhibit similarities in inflammatory tissues and the spinal cord.^37^^,^^38^ Several studies suggest MBCT can reduce pain in people with various chronic pain conditions.^30^^,^^39^, ^40^, ^41^ Our findings confirm that MBCT can help relieve itching in patients with psoriasis.

The online MBCT intervention in our study demonstrated feasibility and potential benefits for psychological outcomes in psoriasis, including applicability in remote or resource-limited areas. In addition, viable online MBCT strategies can be transferred to other developing countries, where physicians often gave no care to patients' mental health.^42^ Future research will assess its cost-effectiveness.

However, our study has several limitations. This study was conducted at a single center. Further research including patients from different regions, diverse ethnic backgrounds, and international populations is needed to improve generalizability. Moreover, most participants had mild-to-moderate psoriasis. Individuals with severe psoriasis, who often experience a heavier psychological burden^43^^,^^44^ may derive even greater benefit from MBCT. Extending follow-up periods will also be important to better capture the relapsing–remitting nature of psoriasis. Well-designed studies with longer observation windows and parallel-group designs are needed to evaluate long-term efficacy, relapse reduction, and disease recurrence.

In conclusion, this study provides preliminary evidence that online MBCT may represent a standardized and reproducible approach to supporting psoriasis management, particularly in resource-limited settings. These findings cautiously support its use as an adjunctive strategy to potentially enhance patient well-being in psoriasis care.

## Conflicts of interest

None disclosed.
